# Use of the tetrazolium salt MTT to measure cell viability effects of the bacterial antagonist *Lysobacter enzymogenes* on the filamentous fungus *Cryphonectria parasitica*

**DOI:** 10.1007/s10482-013-9907-3

**Published:** 2013-03-26

**Authors:** Nrupali Patel, Peter V. Oudemans, Bradley I. Hillman, Donald Y. Kobayashi

**Affiliations:** Department of Plant Biology & Pathology, School of Environmental and Biological Sciences, Foran Hall, Rutgers, The State University of New Jersey, 59 Dudley Road, New Brunswick, NJ 08901-8520 USA

**Keywords:** Bacterial/fungal interaction, Viability stain, Antifungal, Antagonism, Biological control

## Abstract

Despite substantial interest investigating bacterial mechanisms of fungal growth inhibition, there are few methods available that quantify fungal cell death during direct interactions with bacteria. Here we describe an in vitro cell suspension assay using the tetrazolium salt MTT as a viability stain to assess direct effects of the bacterial antagonist *Lysobacter enzymogenes* on hyphal cells of the filamentous fungus *Cryphonectria parasitica.* The effects of bacterial cell density, fungal age and the physiological state of fungal mycelia on fungal cell viability were evaluated. As expected, increased bacterial cell density correlated with reduced fungal cell viability over time. Bacterial effects on fungal cell viability were influenced by both age and physiological state of the fungal mycelium. Cells obtained from 1-week-old mycelia lost viability faster compared with those from 2-week-old mycelia. Likewise, hyphal cells obtained from the lower layer of the mycelial pellicle lost viability more quickly compared with cells from the upper layer of the mycelial pellicle. Fungal cell viability was compared between interactions with *L. enzymogenes* wildtype strain C3 and a mutant strain, DCA, which was previously demonstrated to lack in vitro antifungal activity. Addition of antibiotics eliminated contributions to MTT-formazan production by bacterial cells, but not by fungal cells, demonstrating that mutant strain DCA had lost complete capacity to reduce fungal cell viability. These results indicate this cell suspension assay can be used to quantify bacterial effects on fungal cells, thus providing a reliable method to differentiate strains during bacterial/fungal interactions.

## Introduction

Understanding interactions between bacteria and fungi have been the topic of interest for years, with the vast majority of studies focusing on characterizing bacteria as biological control agents of plant diseases. While there remains substantial interest in these interactions, especially as they relate to horticulture and agriculture, recent studies investigating interactions with respect to soil ecology and human health (Partida-Martinez and Hertweck 2005; Leveau and Preston 2008; McAlester et al. 2008; Kobayashi and Crouch 2009; Mela et al. 2011) highlight broadening recognition of their relevance. Such increased interest underscores the need to develop methods that can quantitatively evaluate the outcomes of interactions between bacteria and fungi.

Many traditional methods for evaluating these interactions have relied on direct measurements of antifungal activity expressed by bacteria. These often involve standard in vitro assays such as measuring zones of growth inhibition resulting from co-incubation of bacterial and fungal cells on agar media (Kobayashi et al. 2005; Kobayashi and Yuen 2005), or evaluating inhibition of spore germination (Zhang and Yuen 2000). Since many antagonistic bacteria have been characterized specifically as biological control agents of plant diseases, measuring suppression of plant disease has provided an alternative, indirect correlative method for measuring outcomes of bacteria/fungal interactions (Zhang and Yuen 2000; Folman et al. 2003; Kobayashi and Yuen 2005). While these types of methods have been used effectively to quantify gross bacterial effects on fungal growth properties and biocontrol, there is a need to develop additional methods that can rapidly measure direct killing effects of bacteria on fungal cells, especially when evaluating effects and contributing roles of specific bacterial traits on fungal cell viability.

Viability stains have proven useful for evaluating fungal cell responses to various stress conditions. There are a variety of fluorescent dyes that are useful for evaluating cell viability (Hickey et al. 2004), and have been used to investigate fungal interactions with other microbes (Bertaux et al. 2003; Hua et al. 2011). While utility of these stains has provided useful qualitative characterization of interactions, reliance on elaborate equipment, appropriate technical skills and expense of material supplies makes them much less practical as a standard method for quantifying cell viability. In contrast, relatively inexpensive tetrazolium salt stains, such as 3-(4,5-dimethylthiazol-2-yl)-2,5-diphenyl tetrazolium bromide (MTT), have been utilized successfully to quantify effects of antifungal agents on cell viability of a number of fungal species (Levitz and Diamond 1985; Hidore et al. 1991; Pittis and Shattock 1994; Jahn et al. 1995; Meshulam et al. 1995; Krishnan et al. 2005; Etxeberria et al. 2011). In the presence of live cells, the yellow-colored salt MTT is converted through dehydrogenase activity to the purple-colored dye MTT-formazan, which can be quantified easily using a spectrophotometer. While MTT has proven useful in these studies to evaluate effects of antifungal compounds, to our knowledge no study has been conducted that utilizes tetrazolium salts to evaluate direct effects of bacteria on fungi.

The soilborne bacterium *Lysobacter enzymogenes* is widely known for its prolific production of lytic enzymes and secondary metabolites (Christensen and Cook 1978; Sullivan et al. 2003). Described as an antagonist of other microbes, several strains have been evaluated for their potential as biocontrol agents on a variety of different plant species (Folman et al. 2003; Kobayashi and Yuen 2005, 2007). Fungal antagonism displayed by *L. enzymogenes* has been predicted to involve mechanisms that include the production of lytic enzymes such as chitinases, β-1,3-glucanases and proteases, and secondary metabolites such as the antibiotic dihydromaltophilin (HSAF) (Kobayashi and Yuen 2007). While these antifungal compounds are known to contribute to the ability of *L. enzymogenes* to inhibit fungal growth in vitro, neither cell killing effects by the bacterium or the roles that specific antifungal factors provide to the bacterium have been quantitatively evaluated during direct interactions with fungal hosts.

Using a cell suspension assay, we demonstrate here that hyphal cells of the filamentous plant pathogenic fungus *Cryphonectria parasitica* are susceptible to reduction in viability, or killing, during direct interactions with *L. enzymogenes* using the viability stain MTT. Both age and physiological state of fungal cells influence sensitivity to killing by the bacterium. Furthermore, the *L. enzymogenes* global regulatory mutant strain DCA, which was previously demonstrated to be reduced in antifungal activity in vitro (Kobayashi et al. 2005; Kobayashi and Yuen 2005), is incapable of causing hyphal cell death. These results demonstrated that MTT was useful for determining the direct killing effect of *L. enzymogenes* on fungal cells, and also for differentiating antagonistic activity between the wildtype strain and an impaired mutant strain of the bacterium.

## Materials and methods

### Strains, growth conditions and media

*Cryphonectria parasitica* EP155 (Hillman et al. 1990) was grown and maintained at room temperature on potato dextrose agar (PDA; Difco). To generate mycelia for all experiments, 50 ml of potato dextrose broth (PDB) in a 250 ml beaker was inoculated with a single PDA plug of *C. parasitica* and grown for 1 week at room temperature in the dark to minimize pigmentation. *L. enzymogenes* strains C3 (Sullivan et al. 2003) and DCA (Kobayashi et al. 2005) were maintained on 10 % tryptic soy agar (TSA). For all experiments, bacterial strains were grown in 50 ml LB broth (Difco) at 30 °C with shaking overnight.

### Fungal cell viability assay conditions

Mycelial pellicle of *C. parasitica* grown for 1 week in PDB consisted typically of a hardened, compact upper layer and a lower layer comprised of loose filamentous hyphal growth. Unless otherwise indicated, the hardened upper layer was discarded after separation from the lower layer using a spatula. Use of pigmented mycelium was also avoided. The lower layer of the fungal mycelium was harvested by placing onto sterile cheese cloth, and rinsing with 50 ml of 10 mM NaPO_4_ pH 7.0 buffer (PB) to remove residual media. Mycelia were partitioned into 0.1–0.3 gm pieces, placed into 50 ml beakers, and the wet weight determined. Mycelial samples were then inoculated with 3 ml of bacterial suspension and incubated at room temperature. Untreated mycelial controls were inoculated with 3 ml of PB.

To prepare bacterial inocula, *L. enzymogenes* strains were grown in 50 ml LB broth overnight at 30 °C with shaking. Cells were harvested by centrifugation, rinsing once by suspending cells in PB and re-centrifuging before final suspension in PB and adjusting to appropriate densities. For most experiments, the bacterial inoculum was adjusted to a cell density of 1 × 10^9^ cfu/ml. To evaluate the effect of different bacterial cell densities, inocula were adjusted to cell densities of 2 × 10^9^; 1 × 10^9^; 1 × 10^8^; and 1 × 10^7^ cfu/ml.

Treated mycelia were recovered by filtering experimental samples through a 40 µm nylon mesh sterile cell strainer (Fisher Scientific) and rinsing 4 times with 10 ml PB. Mycelial fragments were recovered and suspended in 900 µl of PB, to which 100 µl of MTT solution (5 mg/ml suspended in PB) was added. Samples were incubated in the dark at 30 °C with shaking for 90 min., and then centrifuged for 10 min. The MTT solution was removed completely from the mycelial pellet prior to extracting dye from mycelia with 800 µl of isopropyl alcohol acidified to 0.04 M with HCl and measuring absorbance (A_570_) using a Synergy 2 multi-mode microplate reader (BioTek Instruments). For each sample, absorbance/original mycelial weight was determined and results at each time point were converted to percentages based on absorbance/weight of untreated mycelium at the same time point.

Antibiotic treatment of samples was conducted by adding chloramphenicol (Cm) and gentamycin (Gm) each at 3.6 mg/ml to mycelia samples suspended in PB just prior to addition of MTT. Plating of treated samples to verify cell viability was conducted using replicate treatment samples. Portions of recovered mycelial were plated onto 10 % TSA and observed for the appearance of bacterial and fungal colony growth over the course of 1 week.

### Statistical analysis

**Fig. 1:**
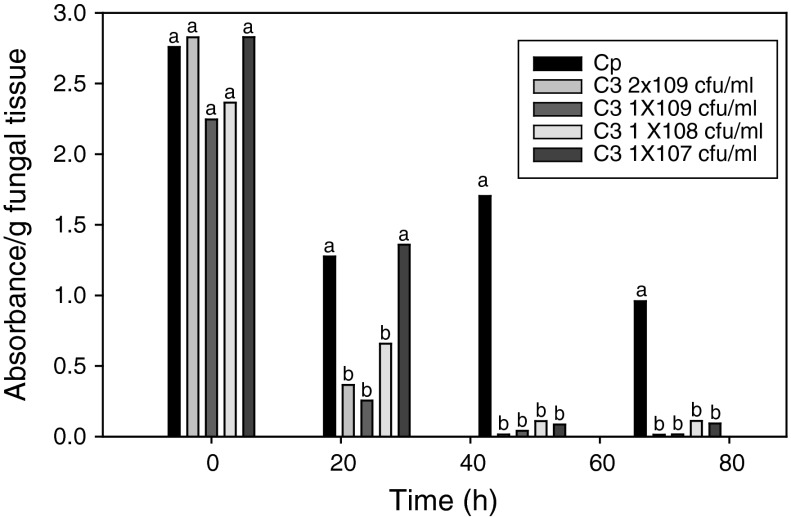
Effect of *Lysobacter enzymogenes* cell density on MTT-formazan production during interactions with *Cryphonectria parasitica*. *Cryphonectria parasitica* treatments: Cp = untreated; C3 2 × 109 cfu/ml = *L. enzymogenes* wt strain C3 cell density of 2 × 10^9^ cfu/ml; C3 1 × 109 cfu/ml = *L. enzymogenes* wt strain C3 cell density of 1 × 10^9^ cfu/ml; C3 1 × 108 cfu/ml = *L. enzymogenes* wt strain C3 cell density of 1 × 10^8^ cfu/ml; C3 1 × 107 cfu/ml = *L. enzymogenes* wt strain C3 cell density of 1 × 10^7^ cfu/ml. Each value represents the mean of 4 replicates. *Like letters* represent no significant difference at each time point according to student Student–Newman–Keuls test (*P* = 0.05)

All experimental treatments were replicated 3–5 times. Data was analyzed using the software program CoStat 4.2 (Cohort Software). With the exception of bacterial inocula density experiment (Fig. 1), all results from treatments were normalized to the control. Analysis of variance was conducted using a completely randomized design and mean separation of treatments was performed using the Student–Newman–Keuls test (*P* = 0.05).

## Results

### Effect of *L. enzymogenes* cell density on fungal cell viability

To determine *L. enzymogenes* effects on fungal cell viability, four different densities of wt strain C3 ranging between 1 × 10^7^ and 2 × 10^9^ cfu/ml were co-incubated with *C. parasitica* mycelia. The effect of different bacterial cell densities on absorbance (A_570_) values resulting from fungal cell production of MTT-formazan was determined by comparing treatments with untreated *C. parasitica* mycelia over a 72 h period. The amount of absorbance/g fungal tissue of MTT-formazan extracted from untreated *C. parasitica* mycelial tissue incubated in a buffered solution declined between 0 and 72 h, but demonstrated that a substantial level of cells remained viable over that time period (Fig. 1). In contrast, absorbance declined rapidly when treated with *L. enzymogenes* cell densities between 1 × 10^8^ and 2 × 10^9^ cfu/ml, where values were reduced to levels below 50 % of the untreated control within 24 h and to nearly undetectable values by 48 h. In comparison, absorbance resulting from production of MTT-formazan by fungal cells treated with the lower bacterial cell density inoculum of 1 × 10^7^ cfu/ml did not decline as rapidly. At 24 h, the absorbance value for this treatment was not significantly different compared with the untreated control. At 48 h co-incubation, however, significant reduction in absorbance values was observed for all samples receiving bacterial treatments compared to the untreated control. At this time point absorbance values were reduced to less than 10 % of that observed with untreated fungal cell samples (Fig. 1).

Visual observations of fungal cell tissue during treatments indicated absorbance values corresponding to high levels of MTT-formazan production also correlated with maintenance of mycelial tissue integrity (data not shown). In contrast, low levels of MTT-formazan production typically correlated with mycelia that no longer remained intact. Individual fungal cell fragments were often observed to be suspended in solution, an observation that corresponded with cell lysis and cell wall degradation.

### Effect of mycelial age on fungal cell viability during interactions with *L. enzymogenes*

Since age can influence cell physiology significantly, especially in terms of preformed mechanisms that function to help resist microbial attack, we wished to determine the effect of fungal mycelial age on cell viability during interactions with *L. enzymogenes.* Therefore, viability of mycelia grown for 2 weeks was compared with that grown for 1 week both alone and during interactions with the bacterium at an inoculum density of 1 × 10^9^ cfu/ml.

Our initial experiment indicated untreated fungal cells produced MTT-formazan and thus remained viable in buffered solution over a 72 h period. To better assess the effects of bacterial treatments at each sampling time point over the 72 h period, we normalized absorbance value results of treated cells to reflect the percentage of values obtained from untreated fungal cells at each sample time point. Since results from initial experiments indicated no differences in absorbance values for MTT-formazan production between bacterial treatments and controls at time 0, experimental sampling was conducted after 24, 48 and 72 h incubation.

**Fig. 2:**
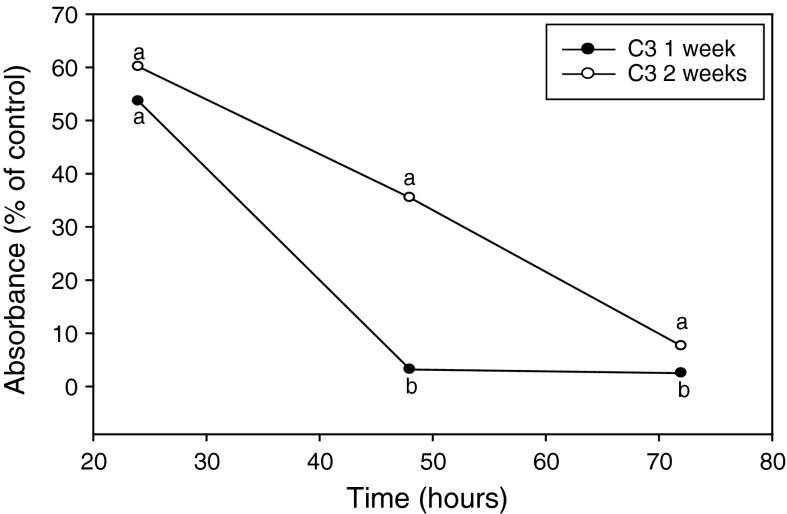
Effect of fungal culture age on MTT-formazan production during interactions between *Lysobacter enzymogenes* and *Cryphonectria parasitica*. Fungal cells were harvested from 1 to 2 week old cultures and treated with *L. enzymogenes* wt strain C3 at an inoculum density of 1 × 10^9^ cfu/ml. Results were normalized and presented as percentages of untreated controls for both culture ages. Each value represents the mean of 4 replicates. *Like letters* represent no significant difference at each time point according to student Student–Newman–Keuls test (*P* = 0.05)

MTT-formazan production, determined as absorbance/g fungal cells, by untreated 1-week-old mycelia often was consistently greater than that produced by cells from 2-weeks-old mycelia for all three sample times (data not shown). As mentioned above, however, because of observed differences between different aged fungal cells, the effects of *L. enzymogenes* on each fungal cell age were evaluated as the percentage of absorbance/g fungal mycelium of untreated fungal cells of the same age. Analysis in this manner allowed for a relative comparison of the effect the bacterium had over time on total viability of fungal cells at each given age. While absorbance values for MTT-formazan production by fungal cells treated with the bacterium decreased over the 72 h period for both 1 and 2 weeks-old mycelia, the relative percentage of absorbance values for dye extracted from 1 week-old mycelia was significantly less compared with that from cells of 2 weeks-old mycelia at 48 and 72 h (Fig. 2).

Relative absorbance values for cells of 1 week-old mycelia treated with the bacterium reached below 5 % of the untreated control by 48 h. In contrast, percentage of absorbance values for treated fungal cells from 2-weeks-old mycelia was reduced to only 35 % of untreated cells within the same 48 h time period and did not reach a value below 5 % at 72 h. These results indicated that during interactions with *L. enzymogenes*, fungal cells from 2 weeks-old mycelia lost viability less rapidly relative to untreated fungal cells of the same age as compared with cells from 1 week-old mycelia.

### Effect of mycelial tissue type on fungal cell viability during interactions with *L. enzymogenes*

**Fig. 3:**
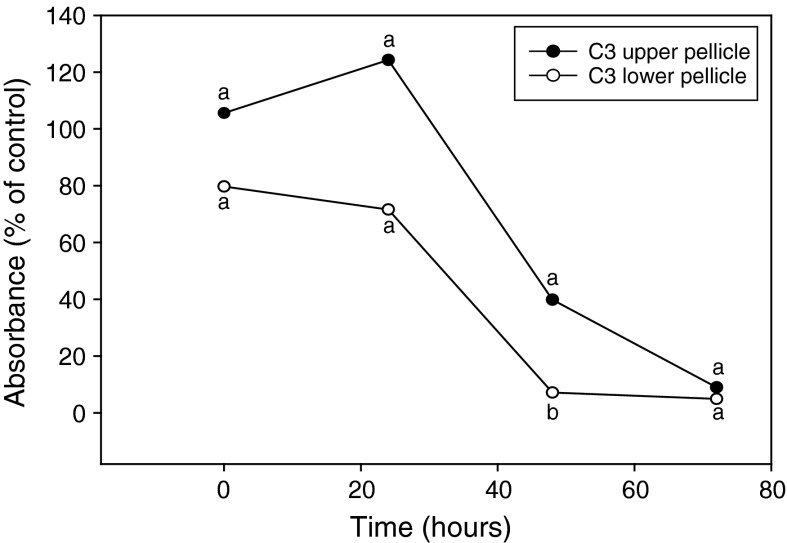
Effect of *Cryphonectria parasitica* mycelium tissue type on MTT-formazan production during interactions between *Lysobacter enzymogenes*. *Cryphonectria parasitica* cells from the hardened upper layer of mycelial pellicle growth were separated from cells comprising the loose matrix of cells of the lower layer and separately treated with *L. enzymogenes* wt strain C3 at an inoculum density of 1 × 10^9^ cfu/ml. Results were normalized and presented as percentages of untreated controls for both pellicle tissue types. Each value represents the mean of 4 replicates. *Like letters* represent no significant difference at each time point according to student Student–Newman–Keuls test (*P* = 0.05)

Since the effect of *L. enzymogenes* on fungal cell viability was influenced by mycelial age, we wanted to assess whether viability of cells obtained from different parts of mycelial pellicle growth was also differentially influenced by the bacterium. Growth of *C. parasitica* after 1 week in PDB consistently formed a hardened, densely packed fungal cell layer as part of the top layer of the mycelial pellicle, and a more loosely packed cell matrix that comprised the lower layer. To evaluate differences between the two mycelial tissue types, the upper and lower layers of mycelial growth were partitioned and treated with *L. enzymogenes* wt strain C3 at an inoculum density of 1 × 10^9^ cfu/ml over a 72 h period. Absorbance values for fungal cells from untreated hardened upper layer of the pellicle were greater at all three time points of 24, 48 and 72 h compared to loosely packed cells from the lower layer (data not shown). To assess fungal cell viability after treatment with *L. enzymogenes*, cells from both mycelial tissue types treated with the bacterial wt strain C3 were compared to the untreated *C. parasitica* cells from the same mycelial tissue type at each sampled time period. Treatment with the bacterium resulted in reduced absorbance values for MTT-formazan production by cells originating from both the upper and lower layers of the mycelial pellicle over the 72 h period (Fig. 3). At 48 h, the relative percentage value of absorbance/g fungal cell wt (Fig. 3) for MTT-formazan produced by cells from the hardened upper layer of the pellicle was significantly greater than that produced by cells from the lower layer. At this time point, absorbance values for MTT-formazan generated by lower layer cells reached below 10 % of the untreated control, while cells from the upper layer remained close to 40 % of the untreated control at the same time period.

### Comparison of *L. enzymogenes* wt strain C3 with the global regulatory mutant strain DCA on fungal cell viability

*Lysobacter enzymogenes* mutant strain DCA contains a disruption in the global regulator gene *clp*, a member of the Crp family of global regulators (Kobayashi et al. 2005). This strain is deficient in the ability to produce antifungal compounds such as lytic enzymes and the major antibiotic dihydromaltophilin (HSAF). It has also lost substantial microbial antagonism activity, as detected by in vitro interaction assays and through evaluation of biological control of plant diseases (Kobayashi et al. 2005; Kobayashi and Yuen 2005; Li et al. 2008). To further demonstrate the differences between the wt strain C3 and the non-antagonist mutant strain DCA, *C. parasitica* cells were inoculated with each bacterial strain at a cell density of 2 × 10^8^ cfu/ml. The lower bacterial cell dosage was chosen as opposed to higher cell dosages used in previous experiments in efforts to prolong fungal cell killing, with the intention of further accentuating differences observed when comparing the wt and mutant strains DCA.

**Fig. 4:**
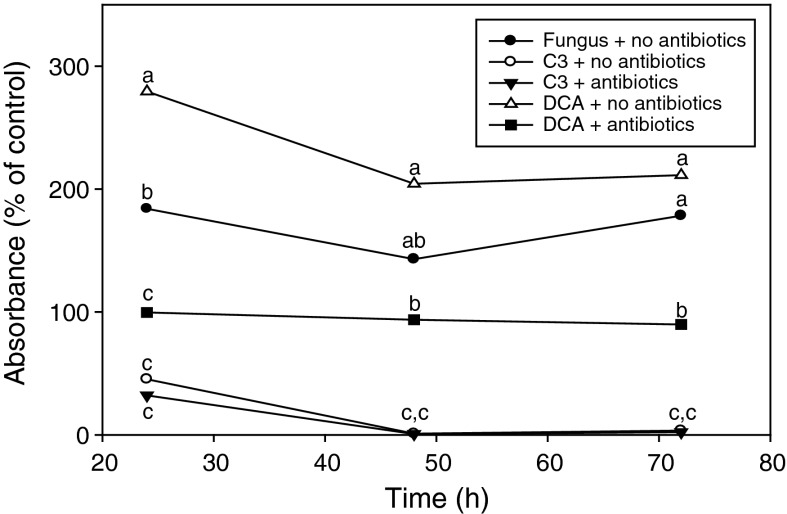
Comparison on *Lysobacter enzymogenes* wt strain C3 and avirulent mutant strain DCA on MTT-formazan production during interactions with *Cryphonectria parasitica*. Treatments consisted of bacterial inocula densities of 2 × 10^8^ cfu/ml. Treatment tests consisted of supplementation with and without antibiotics chloramphenicol (3.6 mg/ml) and gentamycin (3.6 mg/ml). Results were normalized and presented as percentages of fungal cell controls that received no bacterial treatment, but received antibiotic supplementation. Each value represents the mean of 4 replicates. *Like letters* represent no significant difference at each time point according to student Student–Newman–Keuls test (*P* = 0.05)

Significant reduction in absorbance values for MTT-formazan production was observed with fungal cells co-incubated with *L. enzymogenes* wt strain C3 compared with the untreated fungal cells, within a 24 h period, and maximum reduction in absorbance was observed by 48 h (Fig. 4, no antibiotic treatments). In contrast, fungal cells co-incubated with mutant strain DCA showed no reduction in absorbance values compared with untreated fungal cells over a 72 h period. Instead, fungal cells treated with mutant strain DCA displayed higher absorbance values for MTT-formazan production compared with untreated fungal cells at all three time points of 24, 48 and 72 h (Fig. 4, no antibiotics treatments). We concluded from these observations that increased absorbance values may have resulted directly from MTT-formazan production resulting from bacteria bound to the fungal cells.

**Fig. 5:**
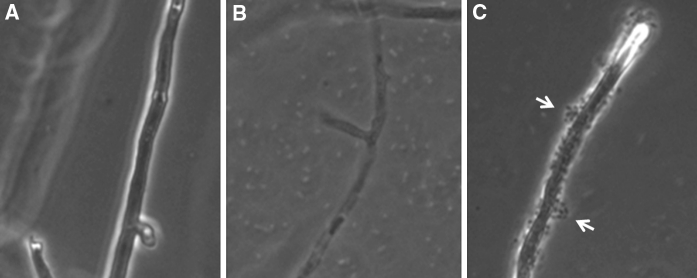
Bright field microscopy of *Cryphonectria parasitica* hyphal cells after a 72 h interaction with *Lysobacter enzymogenes*. **a** Untreated *C. parasitica*; **b** *C. parasitica* treated with *L. enzymogenes* wildtype strain C3. Hyphal cells appear vacuolated and cell walls pitted, and unbound bacterial cells can be detected in the background; **c** *C. parasitica* treated with *L. enzymogenes* mutant strain DCA. *Arrows* depict aggregated bacterial cells bound to hyphal cell

A characteristic feature of *L. enzymogenes* mutant strain DCA is that cells bind non-specifically to surfaces (Kobayashi et al. 2005). Non-specific binding of the mutant bacterial strain to fungal cells was observed over the 72 h period of the interaction assay. In all cases, however, hyphal cells treated with strain DCA remained intact and appeared highly viable. In contrast, the wt strain C3 was no longer observed to be bound to fungal cells at 24 h and remained unbound over the remaining 72 h period, at which time fully intact fungal cells were sparse. Hyphal strands that were recoverable appeared dead or highly stressed as vacuolated, pitted-walled cells (Fig. 5). In order to determine if attached bacteria contributed to MTT-formazan production, the antibiotics Cm (3.6 mg/ml) and Gm (3.6 mg/ml) were added to treatment samples in order to eliminate live bacterial cells. The addition of these antibiotics had an effect on fungal cell viability, since absorbance values for MTT-formazan production by untreated fungal cells receiving no antibiotics were consistently higher compared with those receiving supplementation of Cm and Gm (Fig. 4). The addition of antibiotics also reduced absorbance values for MTT-formazan production by fungal cells treated with mutant strain DCA to more than half the value of those observed with DCA-treated fungal cells that did not receive antibiotic supplementation. However, normalized absorbance values for the strain DCA-treated fungal cell samples supplemented with antibiotics were consistently close to 100 % value that was set for untreated fungal cell supplemented with antibiotics (Fig. 4), suggesting minimal impact of the presence of *L. enzymogenes* mutant strain DCA on fungal cell viability.

In contrast to fungal cells treated with mutant strain DCA, antibiotic supplementation had little effect on the general trend observed with viability of fungal cells treated with *L. enzymogenes* wt strain C3. Absorbance values for MTT-formazan production were reduced in wt strain C3-treated fungal cells within 24 h after inoculation with or without antibiotic supplementation (Fig. 4), with no significant difference between the two different treatments.

To verify viability of fungal and bacterial cells in treatments after supplementation with antibiotics, mycelial fragments were recovered from samples after washing, and plated onto 10 % TSA medium to promote bacterial and fungal cell growth. Consistent fungal colony growth was obtained from plating fungal hyphae recovered from all control treatments that received no bacterial treatment, regardless of whether or not samples were supplemented with antibiotics and regardless of time point (Table 1). As expected, no bacterial growth appeared from fungal control samples receiving no bacterial treatment; however, bacterial growth was observed from fungal cells recovered from treatments with either wt strain C3 or mutant strain DCA, but which did not receive supplementation of antibiotics. In contrast, no bacterial growth was observed from sample treatments receiving antibiotic supplementation, again regardless of time point. These results verified that antibiotic supplementation sufficiently reduced bacterial viability during the MTT assay.

**Table 1:** Colony growth on agar medium resulting from bacterial and fungal cells recovered from interaction samples

Fungal treatment^a^	Interaction sampling time^b^					
	24 h		48 h		72 h	
	B	F	B	F	B	F
No bacteria w/antibiotic	−	+	−	+	−	+
No bacteria w/o antibiotic	−	+	−	+	−	+
C3 w/antibiotic	−	+	−	±	−	−
C3 w/o antibiotic	+	−	+	−	+	−
DCA w/antibiotic	−	+	−	+	−	+
DCA w/o antibiotic^c^	+	+	+	+	+	+

*No bacteria* untreated control, *C3* *L. enzymogenes* wt strain C3, *DCA* *L. enzymogenes* mutant strain DCA

^a^Treatments supplemented with (w/) or without (w/o) the antibiotics gentamycin (3.6 mg/ml) and chloramphenicol (3.6 mg/ml)

^b^Observed growth (+) or no growth (−) of *Lysobacter enzymogenes* (B) and *Cryphonectria parasitica* (F) after plating mycelial fragments recovered from interaction samples on 10 % TSA. ± represents growth in some but not all treatment replicates

^c^For all samples, mixed culture growth of both fungal and bacterial colonies were observed

Fungal growth was observed from plating hyphal cells recovered from treatments with mutant strain DCA, regardless of antibiotic supplementation or time point (Table 1). Plating hyphal cells from samples that did not receive antibiotics resulted in mixed cultures of both bacterial and fungal growth, emphasizing the lack of antifungal activity expressed by mutant strain DCA. Fungal colony growth did not occur from hyphal cells recovered from samples receiving treatment with wt strain C3 with no antibiotic supplementation, regardless of the time point. This observation was a result of the bacterium overgrowing any viable fungal cells on the agar medium. However, supplementation of antibiotics resulted in fungal colony growth occurring from plating of hyphal cells recovered from the 24 h time point and in some recovered from the 48 h time point. No fungal growth was observed when hyphal cells recovered from the 72 h time point were plated. These results corresponded with fungal cell viability assessments based on absorbance values for MTT-formazan production (Fig. 4).

## Discussion

To our knowledge, this is the first study that utilizes the tetrazolium viability stain MTT to demonstrate direct effects of bacteria on fungal cell viability within an in vitro assay system. Fungal cell death during interactions with *L. enzymogenes* was directly correlated over time, concentration of bacteria and physiological state of fungal cells. The assay was also useful for comparing the effects of two different bacterial strains, the wildtype and an impaired mutant strain of *L. enzymogenes*, on fungal cell viability. While this study describes the use of interactions only between *L. enzymogenes* and *C. parasitica*, MTT has proven useful as a viability stain for a broad variety of organisms, including several fungal species. With appropriate alterations in assay conditions, we expect this method to be transferable as a metric for evaluating effects of different bacterial strains on the cell viability of other fungal host systems.

Variations in the physiology of fungal cells, for example by incorporation of pigments into the cell wall (Bell and Wheeler 1986), have long been presumed to function in tolerance to microbial stresses. Results from two of our experiments provide supportive evidence that differences in fungal cell physiology correlate with cell viability in the presence of bacteria. We found that fungal cells originating from younger mycelia lost viability at a higher rate during interactions with *L. enzymogenes* compared with cells from older mycelia (Fig. 2). Similarly, fungal cells originating from loose, hyphal cell matrix found in lower layers of mycelial pellicle growth also lost viability at a higher rate during interactions with the bacterium compared with cells from more dense, hardened mycelial growth found in upper layers of pellicles (Fig. 3). While we do not know the true mechanisms contributing to reduced susceptibility of the fungal cells, we speculate that in both experiments, the physiologically state of cells, the mycelial tissue, or both contributed to reduced susceptibility to cell death caused by the bacterium. It is possible that fungal cells of older age are less susceptible to bacterial attack, due to physiological differences perhaps resulting from cell wall structure, or from the presence of preformed substances that may possess antibacterial activity. We regularly observed that cells within older mycelial tissue and in the hardened upper layer of mycelia consisted of more densely packed cells, which likely precluded exposure to bacterial cells and thus may have reduced the cell death rate caused by the bacterium. Regardless of the mechanism, our results provide strong experimental evidence that supports fungal cell age and mycelial tissue type influence susceptibility to microbial attack.

The *L. enzymogenes* avirulent mutant strain DCA consistently resulted in higher values for MTT-formazan production compared with untreated fungal cell controls over a 72 h period (Fig. 4). Based on this observation, we hypothesized that bacterial cells that remained attached to fungal cells were responsible for the higher MTT-formazan values, since bacteria are known to reduce tetrazolium salts (Tachon et al. 2009; Wang et al. 2010). The addition of antibiotics to treatments eliminated live bacteria (Table 1), and thus their apparent contribution to MTT-formazan production (Fig. 4), supporting the likelihood that bacteria attached to fungal cells contributed to increased production of MTT-formazan.

We observed a correlation between mycelial tissue integrity and fungal cell viability, as well as with bacterial cell attachment (data not shown). The integrity of mycelial tissue was typically reduced upon reduced cell viability, presumably as a result of cell wall degradation by the presence of bacteria. In all cases, once mycelial tissue integrity was lost, bacterial cells were no longer observed to be bound to fungal cells. Our interpretation of these observations is that binding of bacteria to fungal cells represents an early stage of the interaction; however, as the interaction progresses, fungal cell death commences and bacterial binding to fungal cells is substantially reduced.

*Lysobacter enzymogenes* strains are known to produce several factors predicted to contribute to antifungal activity. Examples of such factors include lytic enzymes such as chitinases, β-1,3-glucanases and proteases and the antibiotic dihydromaltophilin (HSAF) (Christensen and Cook 1978; Kobayashi et al. 2005; Yu et al. 2007). In addition, within the genome sequence of *L. enzymogenes* strain C3 are genes encoding several additional factors predicted to contribute to antifungal activity, including those known to contribute to pathogenesis in other bacterial/eukaryotic host systems (Patel and Kobayashi, unpublished data). Despite numerous candidate factors, however, their contributing roles in antagonism towards fungi remain unclear. The method described here provides a quantifiable assay that can help assess these roles, through comparison of mutant strains, during direct interactions with fungal cells and thus providing further insight into mechanisms of antagonism.

## References

[CR1] Bell AA, Wheeler MH (1986). Biosynthesis and functions of fungal melanins. Annu Rev Phytopathol.

[CR2] Bertaux J, Schmid M, Prevost-Boure NC, Churin JL, Hartmann A, Garbaye J, Frey-Klett P (2003) In situ identification of intracellular bacteria related to *Paenibacillus* spp. in the mycelium of the ectomycorrhizal fungus *Laccaria bicolor* S238N. Appl Environ Microbiol 69(7):4243–4248 10.1128/AEM.69.7.4243-4248.2003 PMC165139 12839806

[CR3] Christensen P, Cook FD (1978). *Lysobacter*, a new genus of nonfruiting, gliding bacteria with a high base ratio. Int J Syst Bacteriol.

[CR4] Etxeberria A, Mendarte S, Larregla S (2011). Determination of viability of *Phytophthora capsici* oospores with the tetrazolium bromide staining test versus a plasmolysis method. Rev Iberoam Micol.

[CR5] Folman LB, Postma J, Van Veen JA (2003). Characterisation of *Lysobacter enzymogenes* (Christensen and Cook 1978) strain 3.1T8, a powerful antagonist of fungal diseases of cucumber. Microbiol Res.

[CR6] Hickey PC, Swift SR, Roca MG, Read ND (2004) Live-cell imaging of filamentous fungi using vital fluorescent dyes and confocal microscopy. In: Savidge T, Charalabos P (eds) Methods in microbiology, vol 34. Academic, New York, pp 63–87

[CR7] Hidore MR, Nabavi N, Sonleitner F, Murphy JW (1991). Murine natural killer cells are fungicidal to *Cryptococcus neoformans*. Infect Immun.

[CR8] Hillman BI, Shapira R, Nuss DL (1990). Hypoviulence-associated suppression of host functions in *Cryphonectria parasitica* can be partially relived by high light intensity. Phytopathology.

[CR9] Hua S, Brandl M, Hernlem B, Eng J, Sarreal S (2011). Fluorescent viability stains to probe the metabolic status of aflatoxigenic fungus in dual culture of *Aspergillus flavus* and *Pichia anomala*. Mycopathologia.

[CR10] Jahn B, Martin E, Stueben A, Bhakdi S (1995). Susceptibility testing of *Candida albicans* and *Aspergillus* species by a simple microtiter menadione-augmented 3-(4,5-dimethyl-2-thiazolyl)-2,5-diphenyl-2*H*-tetrazolium bromide assay. J Clin Microbiol.

[CR11] Kobayashi DY, Crouch JA (2009). Bacterial–fungal interactions: from pathogens to mutualistic endosymbionts. Annu Rev Phytopathol.

[CR12] Kobayashi DY, Yuen GY (2005). The role of *clp*-regulated factors in antagonism against *Magnaporthe poae* and biological control of summer patch disease of Kentucky bluegrass by *Lysobacter enzymogenes* C3. Can J Microbiol.

[CR13] Kobayashi DY, Yuen GY (2007). The potential of *Lysobacter* spp. as bacterial biological control agents for plant diseases. CAB Rev Perspect Agric Vet Sci Nutr Nat Res.

[CR14] Kobayashi DY, Reedy RM, Palumbo JD, Zhou J-M, Yuen GY (2005). A *clp* gene homologue belonging to the Crp gene family globally regulates lytic enzyme production, antimicrobial activity and biological control activity expressed by *Lysobacter enzymogenes* strain C3. Appl Environ Microbiol.

[CR15] Krishnan S, Manavathu EK, Chandrasekar PH (2005). A comparative study of fungicidal activities of voriconazole and amphotericin B against hyphae of *Aspergillus fumigatus*. J Antimicrob Chemother.

[CR16] Leveau JHJ, Preston GM (2008). Bacterial mycophagy: definition and diagnosis of a unique bacterial–fungal interaction. New Phytol.

[CR17] Levitz SM, Diamond RD (1985). A rapid colorimetric assay of fungal viability with the tetrazolium salt MTT. J Infect Dis.

[CR18] Li S, Jochum CC, Yu F, Zaleta-Rivera K, Du L, Harris SD, Yuen GY (2008). An antibiotic complex from *Lysobacter enzymogenes* strain C3: antimicrobial activity and role in plant disease control. Phytopathology.

[CR19] McAlester G, O’Gara F, Morrissey JP (2008). Signal-mediated interactions between *Pseudomonas aeruginosa* and *Candida albicans*. J Med Microbiol.

[CR20] Mela F, Fritsche K, de Boer W, van Veen JA, de Graaff LH, van den Berg M, Leveau JHJ (2011). Dual transcriptional profiling of a bacterial/fungal confrontation: *Collimonas fungivorans* versus *Aspergillus niger*. ISME J.

[CR21] Meshulam T, Levitz SM, Christin L, Diamond RD (1995). A simplified new assay for assessment of fungal cell damage with the tetrazolium dye, (2,3)-bis-(2-methoxy-4-nitro-5-sulphenyl)-2*H*)-tetrazolium-5-carboxanilide (XTT). J Infect Dis.

[CR22] Partida-Martinez LP, Hertweck C (2005). Pathogenic fungus harbours endosymbiotic bacteria for toxin production. Nature.

[CR23] Pittis JE, Shattock RC (1994). Viability, germination and infection potential of oospores of *Phytophthora infestans*. Plant Pathol.

[CR24] Sullivan RF, Holtman MA, Zylstra GJ, White JF, Kobayashi DY (2003). Taxonomic positioning of two biological control agents for plant diseases as *Lysobacter enzymogenes* based on phylogenetic analysis of 16S rDNA, fatty acid composition and phenotypic characteristics. J Appl Microbiol.

[CR25] Tachon S, Michelon D, Chambellon E, Cantonnet M, Mezange C, Henno L, Cachon R, Yvon M (2009). Experimental conditions affect the site of tetrazolium violet reduction in the electron transport chain of *Lactococcus lactis*. Microbiology.

[CR26] Wang H, Cheng H, Wang F, Wei D, Wang X (2010). An improved 3-(4,5-dimethylthiazol-2-yl)-2,5-diphenyl tetrazolium bromide (MTT) reduction assay for evaluating the viability of *Escherichia coli* cells. J Microbiol Methods.

[CR27] Yu F, Zaleta-Rivera K, Zhu X, Huffman J, Millet J, Harris S, Yuen G, Li X, Du L (2007). Structure and biosynthesis of HSAF, a broad spectrum antimycotic with a novel mode of action. Antimicrob Agents Chemother.

[CR28] Zhang Z, Yuen GY (2000). Effects of culture fluids and preinduction of chitinase production on biocontrol of Bipolaris leaf spot by *Stenotrophomonas maltophilia* C3. Biol Control.

